# Assessment of rAAVrh.74.MHCK7.micro-dystrophin Gene Therapy Using Magnetic Resonance Imaging in Children With Duchenne Muscular Dystrophy

**DOI:** 10.1001/jamanetworkopen.2020.31851

**Published:** 2021-01-04

**Authors:** Rebecca J. Willcocks, Sean C. Forbes, Glenn A. Walter, Lee Sweeney, Louise R. Rodino-Klapac, Jerry R. Mendell, Krista Vandenborne

**Affiliations:** 1 Department of Physical Therapy, University of Florida, Gainesville; 2 Sarepta Therapeutics Inc, Cambridge, Massachusetts; 3 Center for Gene Therapy, The Research Institute at Nationwide Children’s Hospital, Columbus, Ohio

## Abstract

This case-control study uses magnetic resonance imaging and spectroscopy to evaluate the association between treatment with recombinant adeno-associated virus serotype rh74 (rAAVrh74) and muscle quality in children with Duchenne muscular dystrophy.

## Introduction

Microdystrophin gene transfer using recombinant adeno-associated virus serotype rh74 (rAAVrh74) driven by a skeletal and cardiac muscle-specific promoter with enhanced cardiac expression (MHCK7) (SRP-9001) showed promise in an open-label gene transfer study in Duchenne muscular dystrophy (DMD), with robust transgene expression on muscle biopsy (74%-96% of fibers).^1^ However, biopsy data reflect a small sample of muscle, whereas muscle quality in large muscle groups can be objectively and noninvasively measured using quantitative magnetic resonance imaging (qMRI) and spectroscopy (qMRS). qMRI and qMRS are powerful tools for monitoring disease progression and therapeutic response in DMD, and they correlate with and identify future loss of ambulatory function.^2,3,4,5^ We hypothesized that qMRI and qMRS data (muscle fat fraction and bulk MRI transverse relaxation time [qT_2_]) would be reduced in 3 boys treated with SRP-9001 compared with an age-matched natural history cohort treated with standard of care and a control group of individuals without DMD.

## Methods

This study was approved by the University of Florida institutional review board, and parents provided written informed consent. This study followed the Strengthening the Reporting of Observational Studies in Epidemiology (STROBE) reporting guideline for case-control studies. In this case-control study, 3 of 4 participants (75%) enrolled in the Systemic Gene Delivery Clinical Trial for Duchenne Muscular Dystrophy (NCT03375164) independently volunteered for qMRI and qMRS evaluation at the University of Florida between 2018 and 2020. These participants were imaged once or twice after systemic delivery of SRP-9001, between 6 and 24 months after treatment, using the MR protocols implemented in the multicenter Magnetic Resonance Imaging and Biomarkers for Muscular Dystrophy (ImagingDMD) study (NCT01484678). A natural history comparison cohort was drawn retrospectively from the ImagingDMD study. We identified 54 participants who had a total of 110 study visits (between 2011 and 2018) aged between 4.9 and 7.9 years, matching the age of the participants who received SRP-9001. All participants in the natural history cohort were treated with corticosteroids, and none of them were treated with dystrophin restoration therapy.^3^ We also retrospectively identified 17 age-matched individuals without DMD for a comparison control cohort. In all participants, qMRI and qMRS was used to measure leg muscle fat fraction and qT_2_, as previously published.^3^ Briefly, we acquired multiecho axial gradient echo images and multiecho spin echo images in the calves and thighs and localized single-voxel proton MRS (1H-MRS) in the vastus lateralis (VL) and soleus muscles.^3^ Data are presented as means and SDs, calculated using Prism version 8.0 (GraphPad). Statistical analysis was not performed due to the small sample.

## Results

**Figure 1.**
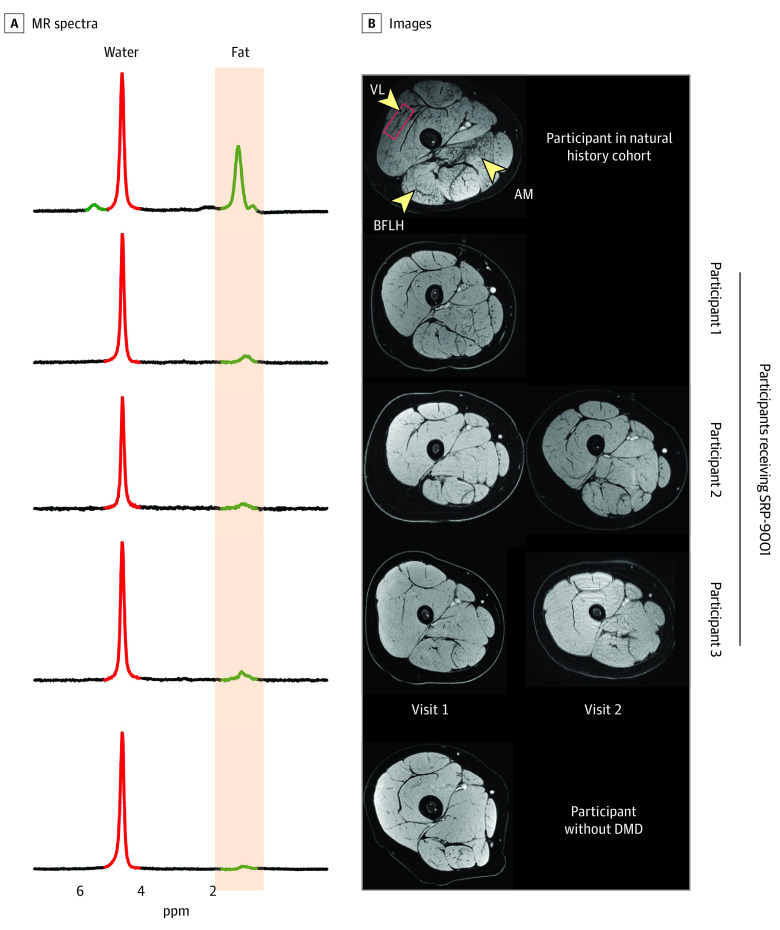
Example Magnetic Resonance (MR) Spectra and Images From Participants in the SRP-9001 Cohort, Natural History Cohort, and Control Cohort Spectra (A) and images (B) of representative participants from the natural history cohort, each participant who received SRP-9001 (participant 1, scan taken 18 months after treatment; participant 2, scans taken 6 and 18 months after treatment; participant 3, scans taken at 12 and 24 months after treatment), and a participant from the control cohort. AM indicates adductor magnus; BFLH, biceps femoris long head; DMD, Duchenne muscular dystrophy; and VL, vastus lateralis.

**Figure 2.**
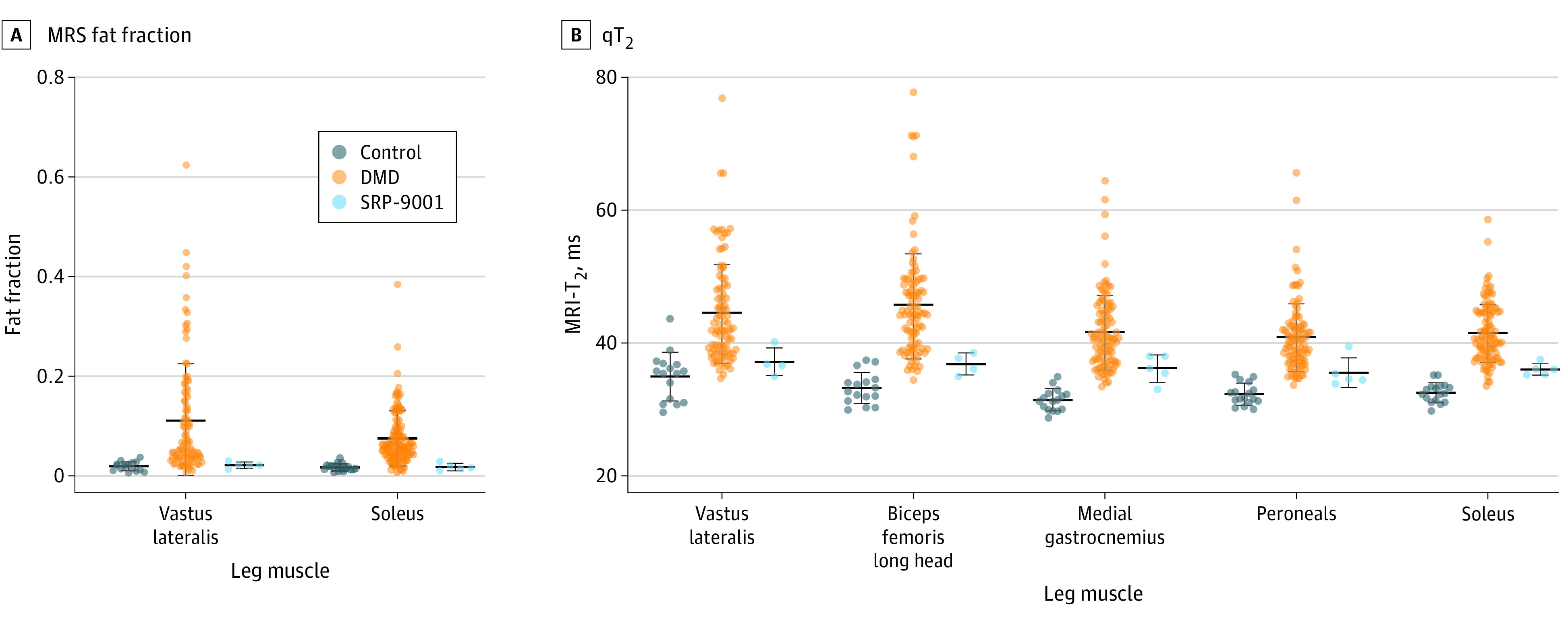
Fat Fraction and Transverse Relaxation Time (qT_2_) Across Multiple Muscles Based on Quantitative Magnetic Resonance Imaging (MRI) and Spectrometry (MRS) The fat fraction (A) and qT_2_ (B) were lower in participants in the SRP-9001 cohort across multiple leg muscles compared with those in the natural history cohort. Due to motion artifact, no upper leg qT_2_ data were available for 1 participant in the SRP-9001 cohort. Although statistical analysis was not performed, group mean data reflected lower quantitative MR biomarker values in the SRP-9001 cohort. Dots indicate individual data points; center lines, means; whiskers, SDs.

Raw MR images acquired in the 3 boys who received SRP-9001 (mean [SD] age, 6.8 [1.0] years; 5 data points) and a representative image for the 54 participants in the natural history cohort (mean [SD] age, 6.8 [0.7] years) and 17 participants in the control cohort (mean [SD] age, 6.7 [0.8] years) are shown in Figure 1. Minimal fat infiltration was observed on MR images from the participants who received SRP-9001 compared with the participants from the natural history cohort, whose muscles have dark patterning throughout, particularly in the biceps femoris long head and adductor magnus. Similarly, spectra from the VL showed a visible fat peak, reflecting accumulation of intramuscular fat (value, 0.13) in the participant from the natural history cohort, which was minimal in the control and SRP-9001 cohorts. In the boys who received SRP-9001, mean (SD) VL MRS fat fraction was lower than that found in the natural history cohort, with 100 data points (0.02 [0.01] vs 0.11 [0.11]) and similar to that in the control cohort, with 17 data points (0.02 [0.01]) (Figure 2A). VL fat fraction was stable over 12 months (the time between MR visits) in 2 boys who received SRP-9001 and had repeated measurements (mean [SD] change 0.00 [0.01]) but not among the 42 participants in the natural history cohort with repeated measurements (mean [SD] change, 0.05 [0.07]). qT_2_ across 5 upper and lower leg muscles was greater in the natural history cohort than in the SRP-9001 or control cohorts, as shown in Figure 2B (eg, mean [SD] qT_2_ for VL: natural history, 44.7 [7.7] ms; SRP-9001, 37.3 [2.2] ms; control, 35.1 [3.7] ms).

## Discussion

These MR data indicate marked sparing and minimal fat infiltration in boys with DMD who received SRP-9001 compared with an age-matched natural history cohort. qMRI and qMRS biomarkers are valuable adjuncts to clinical assessments because of their sensitivity to subclinical disease progression and lack of dependence on participant growth, maturation, and motivation.^3^ In 3 boys with DMD who received SRP-9001, VL fat fraction, which is associated with loss of ambulation, was within unaffected control ranges up to 24 months after gene therapy. qT_2_, which is influenced by inflammation and fat infiltration, was also lower in the boys who received SRP-9001 than those in the natural history cohort. These data support previously published histological and functional evidence for milder disease involvement following gene replacement in DMD.^1^ However, study limitations, including small sample size and lack of MR data before gene therapy, warrant further investigation. The MR results in this report also demonstrate the value of noninvasive qMRI and qMRS measures in examining the efficacy of novel therapeutics in clinical trials in young boys with DMD.
